# Utilizing neurodegenerative markers for the diagnostic evaluation of amyotrophic lateral sclerosis

**DOI:** 10.1186/s40001-023-01596-4

**Published:** 2024-01-06

**Authors:** Kateřina Klíčová, Jan Mareš, Kateřina Menšíková, Michaela Kaiserová, David Friedecký, Petr Kaňovský, Miroslav Strnad, Radoslav Matěj

**Affiliations:** 1 Department of Neurology, Faculty of Medicine and Dentistry, Palacky University and University Hospital Olomouc, Olomouc, Czech Republic; 2 Laboratory of Inherited Metabolic Disorders, Faculty of Medicine and Dentistry, Palacky University and University Hospital Olomouc, Olomouc, Czech Republic Olomouc Czech Republic; 3 Laboratory of Growth Regulators, Faculty of Science and Institute of Experimental Botany of the Czech Academy of Sciences, Palacky University Olomouc, Olomouc, Czech Republic; 4 Department of Pathology and Molecular Medicine, Third Faculty of Medicine, Charles University and Thomayer University Hospital, Prague, Czech Republic

**Keywords:** Amyotrophic lateral sclerosis, Cerebrospinal fluid, Biomarkers

## Abstract

**Background:**

Amyotrophic lateral sclerosis (ALS) is a neurodegenerative disorder characterized by progressive deterioration of upper and lower motor neurons. A definitive diagnostic test or biomarker for ALS is currently unavailable, leading to a diagnostic delay following the onset of initial symptoms. Our study focused on cerebrospinal fluid (CSF) concentrations of clusterin, tau protein, phosphorylated tau protein, and beta-amyloid1–42 in ALS patients and a control group.

**Methods:**

Our study involved 54 ALS patients and 58 control subjects. Among the ALS patients, 14 presented with bulbar-onset ALS, and 40 with limb-onset ALS. We quantified biomarker levels using enzyme-linked immunosorbent assay (ELISA) and compared the results using the Mann–Whitney U-test.

**Results:**

Significant elevations in neurodegenerative markers, including tau protein (*p* < 0.0001), phosphorylated tau protein (*p* < 0.0001), and clusterin (*p* = 0.038), were observed in ALS patients compared to controls. Elevated levels of tau protein and phosphorylated tau protein were also noted in both bulbar and limb-onset ALS patients. However, no significant difference was observed for beta-amyloid1–42. ROC analysis identified tau protein (AUC = 0.767) and p-tau protein (AUC = 0.719) as statistically significant predictors for ALS.

**Conclusion:**

Our study demonstrates that neurodegenerative marker levels indicate an ongoing neurodegenerative process in ALS. Nonetheless, the progression of ALS cannot be predicted solely based on these markers. The discovery of a specific biomarker could potentially complement existing diagnostic criteria for ALS.

## Introduction

Amyotrophic lateral sclerosis (ALS) is a severe, progressive, multisystem degenerative disorder characterized by structural changes affecting both upper and lower motor neurons. ALS, which was first described in 1874, is also commonly referred to as Charcot disease, Lou Gehrig's disease, or motor neuron disease (MND) [1–3]. Individuals with ALS typically manifest symptoms of muscle atrophy, muscle weakness, heightened fatigue, and difficulties in swallowing. The progressive functional deterioration associated with ALS ultimately leads to a loss of self-sufficiency, and a significant number of patients succumb to complications related to the disease, often involving respiratory failure. Despite being relatively rare, ALS imposes a substantial burden on individuals, society, and the economy [4, 5].

Risk factors for ALS include older age, male gender, and a family history of the disease [6]. Incidence and prevalence rates for ALS are on the rise in various regions of the world. In Europe, the estimated annual incidence is 2.2 cases per 100,000 population. Population studies conducted elsewhere have reported lower incidence rates in East Asia, at 0.89 cases per 100,000 population, and in South Asia, at 0.79 cases per 100,000 population per year [7].

Diagnosis of ALS primarily relies on clinical examination and electromyographic examination (EMG) [2, 3]. While the clinical accuracy of ALS diagnosis is generally high, at times, it can be challenging to differentiate ALS from other conditions like Kennedy's disease, myasthenia gravis, cervical spondylotic myelopathy, X-linked spinobulbar muscular atrophy and multifocal motor neuropathy (MMN) [1]. Currently, there is no definitive diagnostic test or biomarker for ALS. The development of novel biomarkers for ALS holds the potential to enhance both the diagnosis and comprehension of the often unpredictable disease progression [8–10]. The introduction of new biomarkers has previously demonstrated its capacity to improve diagnostic and prognostic precision, as evidenced in the case of various other neurodegenerative disorders [11–13].

In our research, our focus was directed towards the assessment of clusterin, tau protein, phosphorylated tau protein, and beta-amyloid1–42 levels within CSF. Clusterin, also known as apolipoprotein J, is a glycoprotein with a molecular weight ranging from 75 to 80 kDa. This multifunctional protein serves as a cytoprotective chaperone and is often referred to as the "guardian of the brain" due to its significant biological role. Clusterin is involved in maintaining protein homeostasis, modulating signal transduction networks, and inhibiting apoptosis. Notably, upregulation of clusterin has been reported in Alzheimer's disease as well as various cancer types [14–17].

Tau protein, a member of the microtubule-associated protein (MAP) family, plays a crucial role in axonal transport and the preservation of neuronal cytoskeleton integrity. Aberrant phosphorylation and hyperphosphorylation of tau protein lead to the formation of insoluble aggregates that disrupt axonal transport, induce microtubule collapse, and result in neuronal dysfunction. Tau protein's involvement is paramount in the pathogenesis of neurodegenerative diseases [18–21].

Beta-amyloid1–42 is produced through the cleavage of the amyloid precursor protein. This specific form, amyloid beta 1–42, is recognized for its significant toxicity and is primarily associated with Alzheimer's disease [22].

Exploring the utilization of both new and existing markers that are associated with other neurodegenerative disorders presents an intriguing strategy for identifying specific markers for the diagnosis of ALS. The quest for novel biomarkers in ALS transcends diagnostic aspects; it also holds broader ramifications and potential to the understanding of the disease itself and the development of therapeutic approaches. The exploration of new biomarkers, may unveil specific molecular and biochemical facets associated with this disease. These biomarkers can serve as indicators of pathological processes underlying the development of ALS.

Beyond diagnostic consequences, the discovery of new biomarkers can furnish crucial insights for designing targeted therapeutic strategies. The identification of biomarkers linked to specific pathological pathways may open avenues for novel treatment approaches and drugs directed at the pathological mechanisms associated with ALS. Overall, the pursuit of new biomarkers in ALS constitutes a complex process with the potential to impact diagnostics, enhance our comprehension of pathogenesis, and contribute to the development of new therapeutic strategies for this serious neurodegenerative condition.

## Methods

### Patients and controls

The study enrolled a cohort of 112 participants, comprising 54 individuals with ALS and 58 individuals in the control group. Among the ALS group, there were 54 patients, consisting of 19 females and 35 males, with ages ranging from 40 to 80 years and a median age of 62. Specifically, 14 ALS patients exhibited bulbar onset symptoms, while 40 experienced limb onset symptoms. In the control group (CG), there were a total of 58 patients, including 26 females and 32 males, with ages ranging from 43 to 83 years and a median age of 60. Three patients within the entire cohort reported a family history of ALS. The average duration from the onset of initial symptoms to the definitive diagnosis was approximately 11.74 months. The detailed data are presented in Table 1.

**Table 1:** Patient´s characteristic

	ALS	CG	*p*
Patients, *n*	54	58	–
ALS onset (limb/bulbar)	40/14	–	–
Gender (male/female)	35/19	32/26	0.338
Age, median (min–max)	62 (40–80)	60 (43–83)	0.120
Diagnostic delay (months)	11.74	–	–
Incidence in the family yes/no	3/51	–	–

The table summarizes the characteristics for ALS patient groups and a healthy control group, including information on the number and proportion of patients, ALS onset, gender, age, familial incidence, and diagnostic delay. The comparison between patients with ALS and control groups revealed no significant differences in terms of age (*p* = 0.120, independent sample t-test) and gender distribution (*p* = 0.338, Fisher's exact test). ALS, amyotrophic lateral sclerosis; CG, control group

Inclusion criteria for participant selection in the study involved a confirmed diagnosis of ALS based on the El Escorial criteria [23] and the absence of any other significant medical conditions. Cerebrospinal fluid samples were collected at diagnosis. The control group consisted of individuals diagnosed with conditions such as tension type headache, lower back pain or dizziness without focal neurological abnormalities, in whom lumbar puncture was performed to exclude the presence of pathological process within the central nervous system. None of the subjects had signs of neurodegenerative disease. CSF was obtained through lumbar puncture while patients were seated, using an atraumatic needle. The puncture was directed to the L4/5 intervertebral space. Subsequently, 10 ml of CSF were collected from each patient into a sterile tube without any additives. The collected samples were morphologically examined and then centrifuged at 1100*g* for 10 min at 4 °C to prepare them for further analysis.

### Biomarkers analysis

The concentrations of individual biomarkers were quantified through ELISA in compliance with the European In Vitro Diagnostic Directive (CE-IVD) using an Atellica® CH analyzer, manufactured by Siemens in the United States.

Clusterin concentration in CSF was determined via sandwich ELISA employing a biotin-labeled antibody provided by Biovendor Laboratory Medicine, Czech Republic, with the designated catalog number RD194034200R. The assay's minimum detectable level for clusterin is 0.50 μg/l, and the CSF samples were subjected to a 100-fold dilution before analysis.

The total soluble tau protein and phosphorylated tau protein (p-tau 181) were assessed using sandwich ELISA kits conforming to the In Vitro Diagnostic Directive (IVD-CE), specifically the Total-tau ELISA by Euroimmun, based in Lubeck, Germany, and bearing the catalog number EQ 6531–9601-L. In this case, the CSF samples were analyzed without dilution.

For the measurement of beta-amyloid1–42, a sandwich ELISA kit also in compliance with the IVD-CE was employed, specifically the beta-amyloid1–42 ELISA by Euroimmun, with catalog number EQ 6521–9601-L. This ELISA involved a solid-phase capture of the amyloid peptide using a monoclonal antibody. CSF samples were added and incubated with a biotinylated antibody, and this complex was subsequently detected using peroxidase-labeled streptavidin. Similar to the tau protein assays, the CSF samples were not subjected to any dilution.

### Statistical analysis

Shapiro–Wilk normality tests showed that the data did not have a normal distribution. Therefore, data were expressed as median, minimum, and maximum value, and independent samples were compared using the Mann–Whitney U-test. All tests were performed at the 0.05 significance level. The statistical software IBM SPSS Statistics for Windows, Version 23.0 was used for statistical processing. Armonk, NY: IBM Corp.

In this investigation, Receiver Operating Characteristic (ROC) analysis was utilized to evaluate the diagnostic efficacy of the method under examination. ROC curves were generated to assess the balance between sensitivity and specificity at various threshold values. The ideal threshold point was identified to maximize diagnostic accuracy. The area under the roc curve (AUC) was computed to quantify the method's overall discriminatory capability. ROC analysis was employed to facilitate the assessment and selection of the most appropriate diagnostic criteria for our study.

## Results

**Fig. 1:**
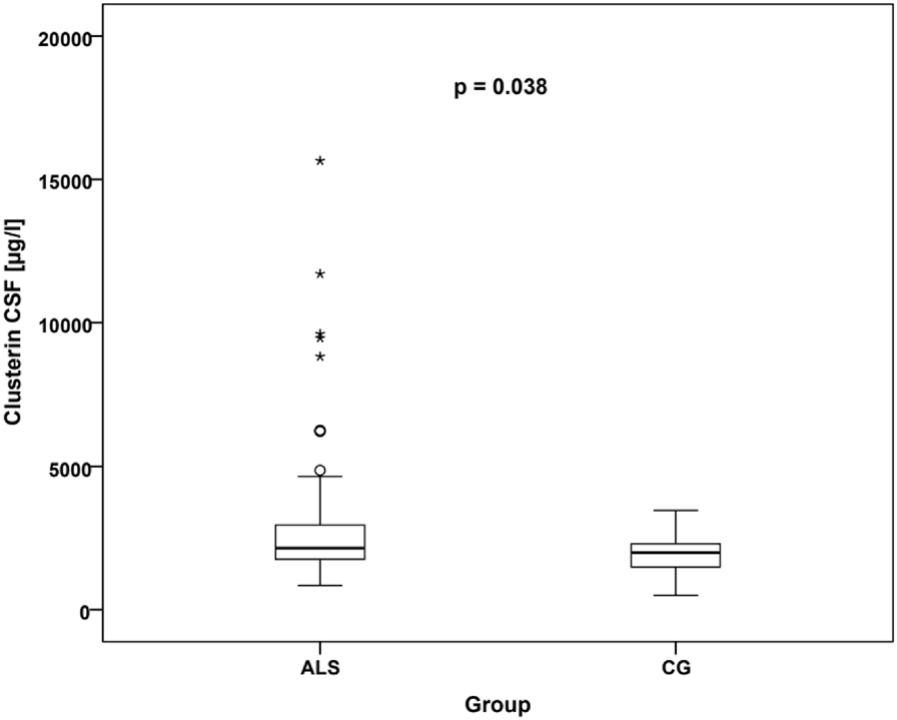
CSF clusterin levels in ALS patients and controls. Comparison of clusterin (µg/l) levels in the CSF between the ALS and control group. The distribution of measured values was represented using box plots (the horizontal line inside the box represents the median, the lower edge of the box represents the first quartile, the upper edge represents the third quartile, whiskers indicate the maximum and minimum measured values, and if outliers were found in the dataset, they are plotted as circles and asterisks). ALS, amyotrophic lateral sclerosis; CG, control group; CSF, cerebrospinal fluid

**Fig. 2:**
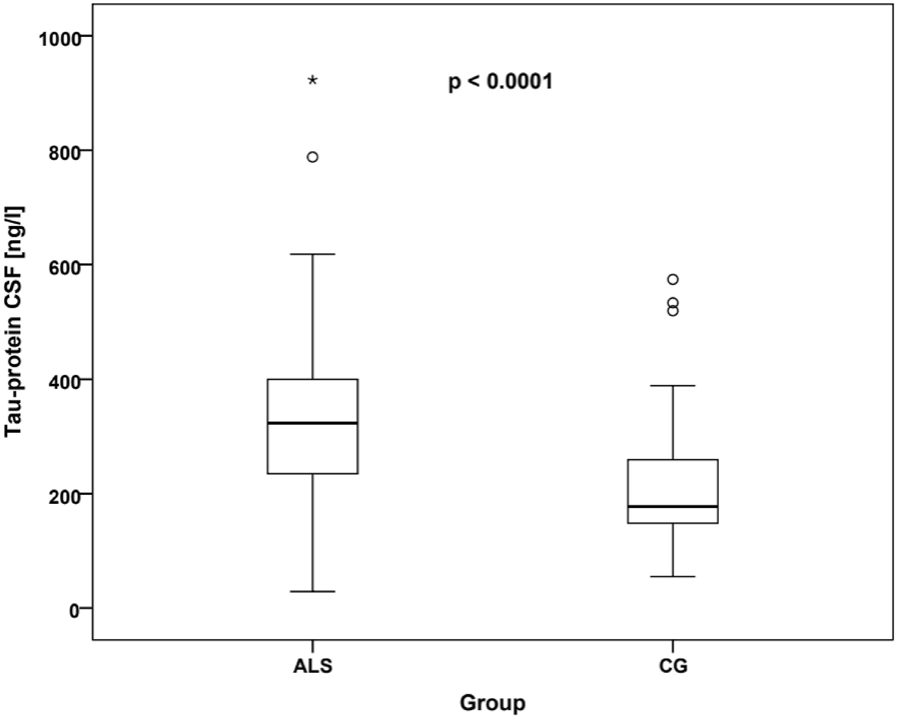
CSF tau protein levels in ALS patients and controls. Comparison of tau protein (ng/l) levels in the CSF between the ALS and control group. The distribution of measured values was represented using box plots (the horizontal line inside the box represents the median, the lower edge of the box represents the first quartile, the upper edge represents the third quartile, whiskers indicate the maximum and minimum measured values, and if outliers were found in the dataset, they are plotted as circles and asterisks). ALS, amyotrophic lateral sclerosis; CG, control group; CSF, cerebrospinal fluid

**Fig. 3:**
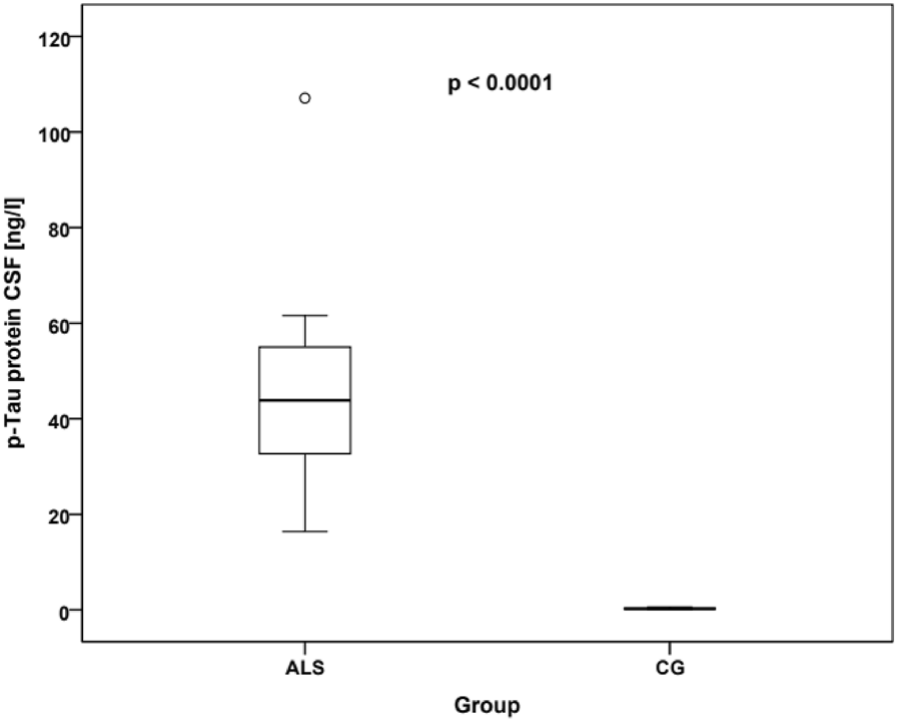
CSF phosphorylated tau protein levels in ALS patients and controls. Comparison of p-tau (ng/l) levels in the CSF between the ALS and control group. The distribution of measured values was represented using box plots (the horizontal line inside the box represents the median, the lower edge of the box represents the first quartile, the upper edge represents the third quartile, whiskers indicate the maximum and minimum measured values, and if outliers were found in the dataset, they are plotted as circles). ALS, amyotrophic lateral sclerosis; CG, control group; CSF, cerebrospinal fluid

The groups of patients with ALS and controls did not significantly differ in terms of age (*p* = 0.120, independent sample t-test) and gender distribution (*p* = 0.338, Fisher's exact test). Following the statistical analysis, it was observed that individuals with ALS exhibited higher levels of clusterin (median 2148 μg/l in ALS vs. median 1987.5 μg/l in the control group; *p* = 0.038, Mann–Whitney U-test), tau protein (median 323.5 ng/l in ALS vs. median 177.5 ng/l in the control group; *p* < 0.0001, Mann–Whitney U-test), and phosphorylated tau protein (median 43.85 ng/l in ALS vs. median 29 ng/l in the control group; *p* = 0.001, Mann–Whitney U-test) in their CSF compared to the control group. However, there were no significant alterations in the levels of beta-amyloid1–42 (median 920.5 ng/l in ALS vs median 918 ng/l in the control group; *p* = 0.774, Mann–Whitney U-test). The respective CSF concentrations of clusterin, tau protein, and phosphorylated tau protein in ALS patients and the control group are illustrated in Figs. 1, 2, and 3. A comprehensive summary of all CSF biomarker levels in ALS patients and controls can be found in Table 2.

**Table 2:** Biomarker levels

	Group						*p*
	ALS			CG			
	Median	Min	Max	Median	Min	Max	
Clusterin CSF [μg/l]	2148	843	15,646	1987.5	500	3460	0.038
Tau-protein CSF [ng/l]	323.5	29	923	177.5	55	574	< 0.0001
Beta-amyloid_1-42_ CSF [ng/l]	920.5	283	1491	918	122	1308	0.774
p-tau protein CSF [ng/l]	43.85	16.40	107.1	29	17	66.3	0.001

The table summarizes statistical comparisons among patients with ALS and a control group. We monitored biomarker levels—clusterin, tau protein, beta-amyloid 1–42, and p-Tau protein in cerebrospinal fluid. The values of significance for the Mann–Whitney U-test are provided in the last column, with tests conducted at a significance level of 0.05

ALS, amyotrophic lateral sclerosis; CG, control group; CSF, cerebrospinal fluid

**Fig. 4:**
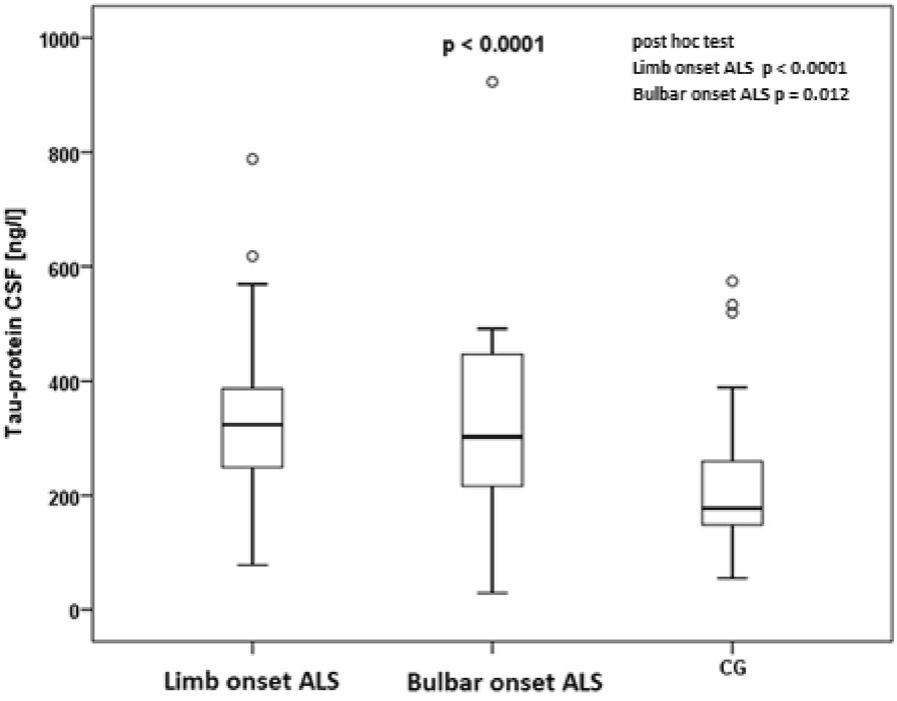
CSF tau protein levels in limb onset of ALS and bulbar onset of ALS patients and controls. Comparison of tau protein levels (ng/l) in cerebrospinal fluid among individuals with limb-onset ALS, bulbar-onset ALS, and the control group. The distribution of measured values was represented using box plots (the horizontal line inside the box represents the median, the lower edge of the box represents the first quartile, the upper edge represents the third quartile, whiskers indicate the maximum and minimum measured values, and if outliers were found in the dataset, they are plotted as circles). ALS, amyotrophic lateral sclerosis; CG, control group, CSF, cerebrospinal fluid

**Fig. 5:**
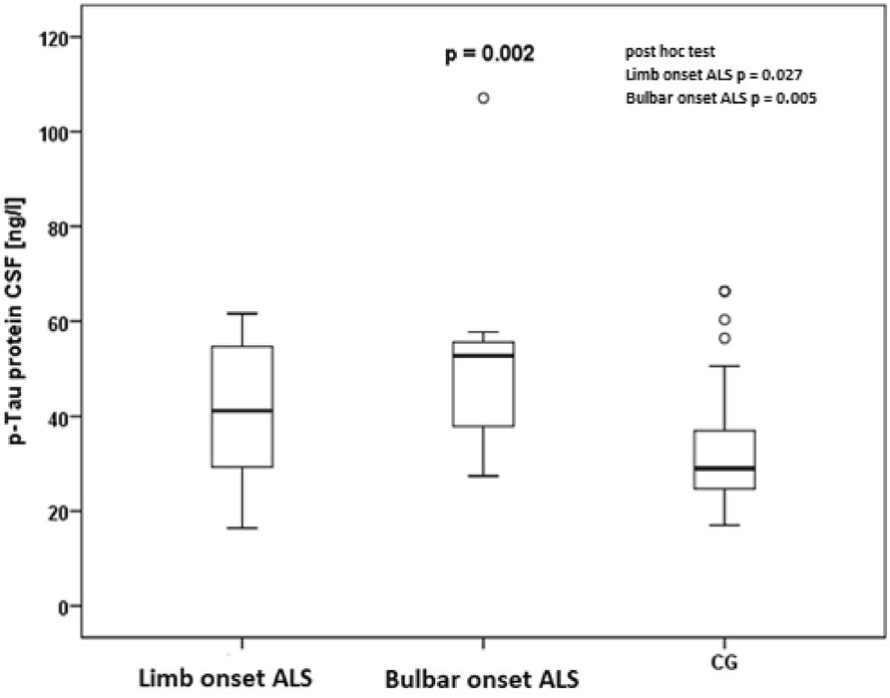
CSF phosphorylated tau protein levels in limb onset of ALS and bulbar onset of ALS patients and controls. Comparison of p-tau protein levels (ng/l) in cerebrospinal fluid among individuals with limb-onset ALS, bulbar-onset ALS, and the control group. The distribution of measured values was represented using box plots (the horizontal line inside the box represents the median, the lower edge of the box represents the first quartile, the upper edge represents the third quartile, whiskers indicate the maximum and minimum measured values, and if outliers were found in the dataset, they are plotted as circles). ALS, amyotrophic lateral sclerosis; CG, control group, CSF, cerebrospinal fluid

During the statistical analysis, it was observed that patients with limb-onset ALS displayed significantly elevated levels of tau protein (median 323.5 ng/l in limb-onset ALS vs. median 177.5 ng/l in the control group; *p* < 0.0001, post hoc test). Similarly, patients with bulbar-onset ALS exhibited higher levels of tau protein (median 302.5 ng/l in bulbar-onset ALS vs. median 177.5 ng/l in the control group; *p* = 0.012, post hoc test). Furthermore, increased levels of phosphorylated tau protein were observed in patients with limb-onset ALS (median 41.2 ng/l in limb-onset ALS vs. median 29 ng/l in the control group; *p* = 0.027, post hoc test) and in those with bulbar-onset ALS (median 52.7 ng/l in bulbar-onset ALS vs. median 29 ng/l in the control group; *p* = 0.005, post hoc test). No significant changes were noted in other parameters. Differences in biomarker levels between ALS cases with limb onset and those with bulbar onset were not found among each other, only in comparison with the control group. The summarized data can be found in Table 3. Additionally, the levels of CSF tau protein and phosphorylated tau protein in limb-onset ALS and bulbar-onset ALS, as well as in the control group, are visually represented in Figs. 4 and 5.

**Table 3:** Limb and bulbar form of ALS, biomarker levels

	Group									*p*	Post hoc test	
	Limb onset ALS			Bulbar onset ALS			CG				*p* (limb vs CG)	*p* (bulbar vs CG)
	Median	Min	Max	Median	Min	Max	Median	Min	Max			
Clusterin CSF [μg/l]	2240	843	11,700	2011.5	1155	15,646	1987.5	500	3460	0,098		
Tau-protein CSF [ng/l]	323.5	78	788	302.5	29	923	177.5	55	574	< 0.0001	< 0.0001	0.012
Beta amyloid_1-42_ CSF [ng/l]	920.5	283	1378	932.5	534.6	1491	918	122	1308	0.959		
p-tau protein CSF [ng/l]	41.2	16.4	61.6	52.7	27.4	107.1	29	17	66.3	0.002	0.027	0.005

The table summarizes statistical comparisons between patients with limb-onset ALS, bulbar-onset ALS, and a control group. Biomarker levels—specifically, clusterin, tau protein, beta-amyloid 1–42, and p-Tau protein—in cerebrospinal fluid were monitored. Comparisons were conducted using the Kruskal–Wallis test. Significant results were observed in the case of tau protein and p-Tau protein, prompting the execution of post hoc tests with Bonferroni correction. No differences in biomarker levels were found between ALS cases with limb onset and with bulbar onset

ALS, amyotrophic lateral sclerosis; CG, control group; CSF, cerebrospinal fluid

**Fig. 6:**
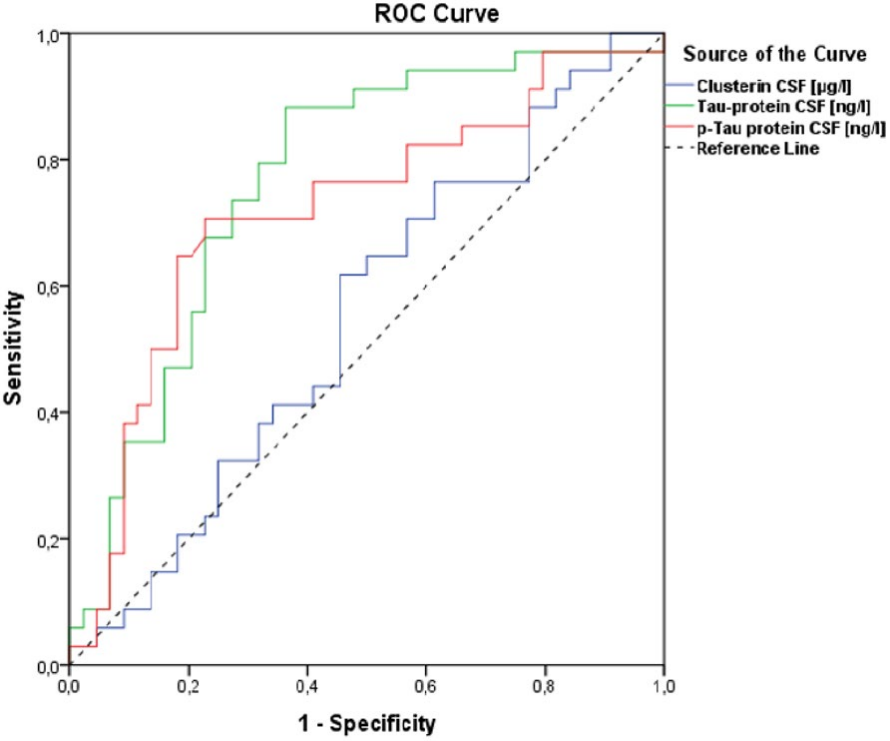
ROC (receiver operating characteristic) curve. The optimal cut-off value for Tau protein in cerebrospinal fluid is 209.5 ng/ml with a sensitivity (SE) of 0.882 and specificity (SP) of 0.636. The optimal cut-off value for p-Tau protein in cerebrospinal fluid is 37.5 ng/l, with a sensitivity of 0.706 and specificity of 0.773

Based on the ROC analysis, the statistically significant predictors for ALS are the CSF levels of tau protein and p-tau protein. In both cases, the AUC falls within the range of 0.7 to 0.8, indicating good discriminatory ability. The optimal cut-off value for tau protein in CSF is determined to be 209.5 ng/ml using the Youden's J statistic, where the sum of sensitivity and specificity is maximized. At this cut-off, the sensitivity (SE) is 0.882, and the specificity (SP) is 0.636 (the test is more sensitive but less specific). The optimal cut-off value for p-tau protein in CSF is found to be 37.5 ng/l, with a sensitivity of 0.706 and a specificity of 0.773. The summarized data can be found in Table 4 and depicted in Fig. 6.

**Table 4:** ROC (reciever operating characteristic) analysis

Test result variable (s)	AUC	*p*	95% CI for AUC	
			Lower bound	Upper bound
Clusterin CSF [μg/l]	0.55	0.45	0.422	0.678
Tau-protein CSF [ng/l]	0.767	0.0001	0.659	0.875
p-tau protein CSF [ng/l]	0.719	0.001	0.6	0.838

The table compiles the results of the conducted ROC analysis, encompassing the area under the curve value, *p*-value, and the lower and upper bounds of the 95% confidence interval for AUC. b. Null hypothesis: true area = 0.5

AUC, Area Under the ROC Curve

## Discussion

Amyotrophic lateral sclerosis is a progressive neurodegenerative disorder primarily diagnosed through clinical history and electromyographic assessments. Currently, there is no definitive diagnostic test or established biomarker for ALS. In our research, our primary objective was to assess the applicability of specific biomarkers in cerebrospinal fluid for the diagnosis of amyotrophic lateral sclerosis. We aimed to investigate whether the analysis of biomarkers could complement the existing diagnostic criteria, potentially enhancing the accuracy of ALS diagnosis. However, promising candidates for diagnostic and prognostic markers include pNF-H, as elevated levels of this marker in CSF have been observed in patients with ALS [26, 27]. In our research, we focused on the use of clusterin, tau protein, phosphorylated tau protein, beta amyloid1-42 and its levels in CSF of patients with ALS.

The chaperone glycoprotein known as clusterin, or apolipoprotein J, plays a significant role in the pathogenesis of neurodegenerative diseases. Clusterin is a heavily glycosylated protein with a molecular weight of 60 kDa, existing as a heterodimer, and it is encoded by the CLU gene. This protein serves as a prevalent extracellular chaperone and has several essential functions in maintaining various physiological processes, including the transport of lipids and tissue remodeling. Clusterin is expressed in various cell types within the nervous system, including neurons, glia, and astrocytes. Its levels in the bloodstream are notably high, typically around 100 μg/ml, while in cerebrospinal fluid, they range from 2–9 μg/ml. Accumulating evidence suggests that clusterin’s role as an extracellular chaperone could directly influence the progression of neurodegenerative diseases [24, 25].

CSF clusterin levels in patients with neurodegenerative disease may aid in diagnosis. The results of a study by Přikrylová Vranová et al. [28] support the role of clusterin in the pathogenesis of Parkinson’s disease. In patients with ALS, an association has been demonstrated between the pathological burden of TDP-43 protein misfolding and cognitive deficits. A study by Gregory et al. (2020) examined the ability to cope with the misfolding of the TDP-43 protein and differences in the expression of protective mechanisms such as clusterin expression. High levels of neuronal clusterin could provide cells with neuroprotection and reduce the clinical manifestations of disease associated with accumulated TDP-43 [29]. In the context of neurodegenerative disease, clusterin levels were also examined by mass spectrophotometry from blood samples, based on the results of which it was possible to distinguish ALS patients with cognitive deficits and controls [30]. The results of our study show that the level of clusterin in CSF is increased in ALS patients compared to CG (ALS median 2148 μg/l vs CG median 1987.5 μg/l; *p* = 0.038 Mann–Whitney U-test). These results suggest the potential of clusterin in the diagnosis of neurodegenerative diseases and highlight its usefulness for the study of ALS. However, CSF levels of clusterin in the limb and bulbar onset of ALS were not significantly different from each other or compared to the control group. The reduced discriminative performance of clusterin analysis may be indicative of variations in biomarker measurements among individual patients. This variability can be attributed to various factors, such as genetic differences or other factors influencing biomarker levels. The diminished discriminative performance of the biomarker may also indicate the need for further research and refinement of diagnostic tools for ALS detection. This may involve exploring new biomarkers, combining multiple biomarkers, or employing additional diagnostic methods.

Tau protein is a protein stabilizing microtubules in axons, it is also important for ensuring axonal transport. Inconsistencies are prevalent among studies related to ALS. In a study by Bourbouli et al. [31], higher levels of tau protein were noted in ALS compared to the control group. In a study by Wilke et al. [32], CSF p-tau was not significantly different in ALS patients compared to control subjects (*p* = 0.287). However, CSF tau was significantly increased (*p* < 0.001). A study by Palladino et al. (2009) found no significant differences in mean CSF tau levels between ALS cases and controls (ALS 126 pg/ml, controls 112 pg/ml), however, in the ALS group, bulbar-onset patients showed elevated CSF tau levels compared to spinal-onset cases. These differences could be related to the older age of patients with bulbar onset. Furthermore, no correlations were found between CSF tau concentrations and rate of disease progression [33]. In our study, we noted significantly increased levels of tau protein (ALS median 323.5 ng/l vs CG median 177.5 ng/l; *p* < 0.0001 Mann–Whitney U-test) and p-tau protein (ALS median 43.85 ng/l vs CG median 29 ng/l; *p* = 0.001 Mann–Whitney U-test) in ALS patients compared to CG. We also noted significantly increased levels of tau protein in bulbar onset of ALS (ALS bulbar onset median 302.5 ng/l in CG median 177.5; *p* = 0.012 post hoc test) and limb onset of ALS (ALS limb onset median 323.5 ng/l vs CG median 177.5 ng/l; *p* < 0.0001 post hoc test) and also p-tau protein in bulbar onset of ALS (ALS bulbar onset median 52.7 ng/l vs CG median 29 ng/l; *p* = 0.005 post hoc test) and limb onset of ALS (ALS limb onset median 41.2 ng/l vs CG median 29 ng/l; *p* = 0.027 post hoc test) compared to the control group. However, no differences were noted between the bulbar and limb onset groups. Despite promising correlations, inconsistencies prevail among studies on CSF tau protein levels in ALS. The reasons for this discrepancy may be diverse, and differences in outcomes may also reflect the biological heterogeneity of ALS [31–33].

Beta-amyloid1–42 is a major component of senile plaques and neurofibrillary tangles, and its accumulation in the brain is thought to be an early toxic event in the pathogenesis of Alzheimer's disease. In a study by Lanznaster et al. [34], beta-amyloid1–42 was increased in a large cohort of ALS patients compared to control subjects (controls 992.9 ± 358.3 ng/l; ALS 1277.0 ± 296, 6 ng/l; *p* < 0.0001). No significant differences were noted in our cohort.

The results of our study suggest elevated levels of clusterin, tau protein, and phosphorylated tau protein in the cerebrospinal fluid of patients with amyotrophic lateral sclerosis. Based on ROC analysis, tau protein and phosphorylated tau protein were identified as significant predictors for ALS. In both cases, the AUC falls within the range of 0.7 to 0.8, indicating a good discriminatory capacity. The optimal cut-off value for CSF tau protein is 209.5 ng/ml (determined using Youden's J statistic; where the sum of sensitivity and specificity is highest, as shown in the table). Sensitivity is 0.882, and specificity is 0.636 (the test is more sensitive but less specific). If the cut-off were adjusted to 247 ng/ml, SE would be 0.735, and SP would be 0.727 (sensitivity and specificity are more balanced). The optimal cut-off value for phosphorylated tau protein in CSF is 37.5 ng/l, resulting in SE = 0.706 and SP = 0.773.

It is worth noting that ALS is one of the most common motor neuron diseases, yet the definitive diagnosis is frequently delayed by approximately one year from the onset of initial symptoms. In our study cohort, the diagnostic delay from the onset of initial symptoms to the establishment of a definitive diagnosis averaged 11.74 months. This delay can be partly attributed to the absence of specific biomarkers that could facilitate the early diagnosis of ALS.

## Conclusion

The identification of biomarkers in CSF in ALS is important for establishing the diagnosis. In the case of ALS, tau protein and p-tau protein could be a useful biomarkers also in the case of differential diagnosis of diseases that manifest similar symptoms. Given that concentration does not correlate with the stage or form of the disease, there is the potential to use it in case of diagnostic doubts at any stage or clinical manifestations.

By simultaneously utilizing multiple markers, we can enhance the diagnostic specificity. While the tau protein demonstrates high sensitivity but low specificity, its integration with p-tau protein can potentially enhance diagnostic capabilities. Additional studies and analyses should also be conducted when combining these markers to determine their diagnostic value. It is crucial to consider that the diagnosis of ALS is intricate, necessitating the integration of clinical, electrophysiological, and laboratory data.

## Data Availability

The data that support the findings of this study are available from the corresponding author, [K.K.], upon reasonable request.

## Abbreviations

ALS: Amyotrophic lateral sclerosis
AUC: Area under the ROC curve
CE-IVD: Compliance with the European In Vitro Diagnostic Directive
CG: Control group
CSF: Cerebrospinal fluid
ELISA: Enzyme-linked immunosorbent assay
EMG: Electromyography
MAP: Microtubule-associated protein
MND: Motor neuron disease
pNF-H: Phosphorylated neurofilament heavy chain
p-tau: Phosphorylated tau protein
ROC: Receiver operating characteristic
SE: Sensitivity
SP: Specificity

## References

[1] Rowland LP, Shneider NA (2001). Amyotrophic lateral sclerosis. N Engl J Med.

[2] Wijesekera LC, Leigh PN (2009). Amyotrophic lateral sclerosis. Orphanet J Rare Dis.

[3] Mathis S, Couratier P, Julian A, Vallat JM, Corcia P, Le Masson G (2017). Management and therapeutic perspectives in amyotrophic lateral sclerosis. Expert Rev Neurother.

[4] Mathis S, Couratier P, Julian A, Corcia P, Le Masson G (2017). Current view and perspectives in amyotrophic lateral sclerosis. Neural Regen Res.

[5] Chiò A, Logroscino G, Traynor BJ, Simeone JC, Goldstein LA, White LA (2013). Global epidemiology of amyotrophic lateral sclerosis: a systematic review of the published literature. Neuroepidemiology.

[6] Ingre C, Ross PM, Piehl F, Kamel F, Fang F (2015). Risk factors for amyotrophic lateral sclerosis. Clin Epidemiol.

[7] Logroscino G, Piccininni M (2019). Amyotrophic lateral sclerosis descriptive epidemiology: the origin of geographic difference. Neuroepidemiology.

[8] Kiernan MC, Vucic S, Cheah BC, Turner MR, Eisen A, Hardiman O, Burrell JR (2011). Zoing MC Amyotrophic lateral sclerosis. Lancet.

[9] Farníková K, Kaňovský P, Nestrašil I, Otruba P (2010). Coexistence of parkinsonism, dementia and upper motor neuron syndrome in four Czech patients. J Neurol Sci.

[10] Menšíková K, Tučková L, Kolařiková K, Bartoníková T, Vodička R, Ehrmann J, Vrtěl R, Procházka M, Kaňovský P, Kovacs GG (2019). Atypical parkinsonism of progressive supranuclear palsy-parkinsonism (PSP-P) phenotype with rare variants in FBXO7 and VPS35 genes associated with Lewy body pathology. Acta Neuropathol.

[11] Feldman EL, Goutman SA, Petri S, Mazzini L, Savelieff MG, Shaw PJ, Sobue G (2022). Amyotrophic lateral sclerosis. Lancet.

[12] Goutman SA, Hardiman O, Al-Chalabi A, Chió A, Savelieff MG, Kiernan MC, Feldman EL (2022). Recent advances in the diagnosis and prognosis of amyotrophic lateral sclerosis. Lancet Neurol.

[13] Peplow PV, Martinez B, Gennarelli TA. Neurodegenerative Diseases Biomarkers. 2022. Neuromethods, sv. 173. s. 155–180. Humana, New York, NY.

[14] Rosenberg ME, Silkensen J (1995). Clusterin: physiologic and pathophysiologic considerations. Int J Biochem Cell Biol.

[15] Jones SE, Jomary C (2002). Clusterin. Int J Biochem Cell Biol.

[16] Nuutinen T, Suuronen T, Kauppinen A, Salminen A (2009). Clusterin: a forgotten player in Alzheimer´s disease. Brain Res Rev.

[17] Wilson MR, Zoubeidi A (2017). Clusterin as a therapeutic target. Expert Opin Ther Targets.

[18] Drubin DG, Kirschner MW (1986). Tau protein function in living cells. J Cell Biol.

[19] Sergeant N, Delacourte A, Buée L (2005). Tau protein as a differential biomarker of tauopathies. Biochim Biophys Acta.

[20] Spillantini MG, Goedert M (1998). Tau protein pathology in neurodegenerative diseases. Trend Neurosci.

[21] Pîrşcoveanu DFV, Pirici I, Tudorică V, Bălşeanu T-A, Albu V-C, Bondari S, Bumbea A-M, Pîrşcoveanu M (2017). Tau protein in neurodegenerative diseases–a review. Rom J Morphol Embryol.

[22] Roher AE, Palmer KC, Yurewicz EC, Ball MJ, Greenberg BD (1993). Morphological and biochemical analyses of amyloid plaque core proteins purified from Alzheimer disease brain tissue. J Neurochem.

[23] Brooks BR, Miller RG, Swash M, Munat TL (2000). El Escorial revisited: revised criteria for the diagnosis of amyotrophic lateral sclerosis. Amyotroph Lateral Scler Other Motor Neuron Disord.

[24] Desikan RS, Thompson WK, Holland D, Hess CP, Brewer JB, Zetterberg H, Blennow K, Andreassen OA, McEvoy LK, Hyman BT, Dale AM (2014). The role of clusterin in amyloid-β-associated neurodegeneration. JAMA Neurol.

[25] Chaplot K, Jarvela TS, Lindberg I (2020). Secreted chaperones in neurodegeneration. Front Aging Neurosci.

[26] Ganesalingam J, An J, Bowser R, Andersen PM, Shaw CE (2013). pNfH is a promising biomarker for ALS. Amyotroph Lateral Scler Frontotemporal Degener.

[27] Kaiserová M, Grambalova Z, Otruba P, Stejskal D, Prikrylova Vranova H, Mares J, Mensikova K, Kanovsky P (2017). Cerebrospinal fluid levels of chromogranin A and phosphorylated neurofilament heavy chain are elevated in amyotrophic lateral sclerosis. Acta Neurol Scand.

[28] Přikrylová Vranová H, Hényková E, Mareš J, Kaiserová M, Menšíková K, Vaštík M, Hluštík P, Zapletalová J, Strnad M, Stejskal D, Kaňovský P (2016). Clusterin CSF levels in differential diagnosis of neurodegenerative disorders. J Neurol Sci.

[29] Gregory JM, Elliot E, McDade K, Bak T, Pal S, Chandran S, Abrahams S, Smith C (2020). Neuronal clusterin expression is associated with cognitive protection in amyotrophic lateral sclerosis. Neuropathol Appl Neurobiol.

[30] Xu Z, Lee A, Nouwens A, Henderson RD, McCombe PA (2018). Mass spectrometry analysis of plasma from amyotrophic lateral sclerosis and control subjects. Amyotroph Lateral Scler Frontotemporal Degener.

[31] Bourbouli M, Rentzos M, Bougea A, Zouvelou V, Constantinides VC, Zaganas I, Evdokimidis I, Kapaki E, Paraskevas GP (2017). Cerebrospinal fluid TAR DNA-binding protein 43 combined with tau proteins as a candidate biomarker for amyotrophic lateral sclerosis and frontotemporal dementia spectrum disorders. Dementia Geriatr Cogn Disord.

[32] Wilke C, Deuschle C, Rattay TW, Maetzler W, Synofzik M (2015). Total tau is increased, but phosphorylated tau not decreased, in cerebrospinal fluid in amyotrophic lateral sclerosis. Neurobiol Aging.

[33] Paladino P, Valentino F, Piccoli T, Piccoli F, La Bella V (2009). Cerebrospinal fluid tau protein is not a biological marker in amyotrophic lateral sclerosis. Eur J Neurol.

[34] Lanznaster D, Hergesheimer RC, Bakkouche SE, Beltran S, Vourc ‘H P, Andres CR, Dufour-Rainfray D, Corcia P, Blasco H (2020). Aβ1–42 and Tau as potential biomarkers for diagnosis and prognosis of amyotrophic lateral sclerosis. Int J Mol Sci.

